# TRF2 as novel marker of tumor response to taxane-based therapy: from mechanistic insight to clinical implication

**DOI:** 10.1186/s13046-024-02998-w

**Published:** 2024-03-09

**Authors:** Sara Iachettini, Irene Terrenato, Manuela Porru, Serena Di Vito, Angela Rizzo, Carmen D’Angelo, Eleonora Petti, Roberto Dinami, Carmen Maresca, Anna Di Benedetto, Aldo Palange, Antonino Mulè, Angela Santoro, Antonella Palazzo, Paola Fuso, Antonella Stoppacciaro, Patrizia Vici, Lorena Filomeno, Francesca Sofia Di Lisa, Teresa Arcuri, Eriseld Krasniqi, Alessandra Fabi, Annamaria Biroccio, Pasquale Zizza

**Affiliations:** 1 IRCCS - Regina Elena National Cancer Institute, Translational Oncology Research Unit, Via Elio Chianesi 53, 00144 Rome, Italy; 2 IRCCS - Regina Elena National Cancer Institute, Clinical Trial Center, Biostatistics and Bioinformatics Unit, Via Elio Chianesi 53, 00144 Rome, Italy; 3 IRCCS - Regina Elena National Cancer Institute, Pathology Unit, Via Elio Chianesi 53, Rome, Italy; 4 Pathology Unit, Fondazione Policlinico Universitario A. Gemelli IRCCS, Rome, Italy; 5 Medical Oncology, Fondazione Policlinico Universitario A. Gemelli IRCCS, Rome, Italy; 6 Department of Woman and Child Health and Public Health, Division of Gynecologic Oncology, Fondazione Policlinico Universitario A. Gemelli IRCCS, Rome, Italy; 7 Department of Clinical and Molecular Medicine, Sant’Andrea Hospital, Sapienza University of Rome, Rome, Italy; 8 IRCCS - Regina Elena National Cancer Institute, Unit of Phase IV Trials, Via Elio Chianesi 53, Rome, Italy; 9 Precision Medicine Unit in Senology, Fondazione Policlinico Universitario A. Gemelli IRCCS, Rome, Italy

**Keywords:** TRF2, Autophagy, Taxanes, Drug sensitivity, TNBC, Predictive marker

## Abstract

**Background:**

Breast Cancer (BC) can be classified, due to its heterogeneity, into multiple subtypes that differ for prognosis and clinical management. Notably, triple negative breast cancer (TNBC) – the most aggressive BC form – is refractory to endocrine and most of the target therapies. In this view, taxane-based therapy still represents the elective strategy for the treatment of this tumor. However, due variability in patients’ response, management of TNBC still represents an unmet medical need.

Telomeric Binding Factor 2 (TRF2), a key regulator of telomere integrity that is over-expressed in several tumors, including TNBC, has been recently found to plays a role in regulating autophagy, a degradative process that is involved in drug detoxification.

Based on these considerations, we pointed, here, at investigating if TRF2, regulating autophagy, can affect tumor sensitivity to therapy.

**Methods:**

Human TNBC cell lines, over-expressing or not TRF2, were subjected to treatment with different taxanes and drug efficacy was tested in terms of autophagic response and cell proliferation. Autophagy was evaluated first biochemically, by measuring the levels of LC3, and then by immunofluorescence analysis of LC3-puncta positive cells. Concerning the proliferation, cells were subjected to colony formation assays associated with western blot and FACS analyses. The obtained results were then confirmed also in mouse models. Finally, the clinical relevance of our findings was established by retrospective analysis on a cohort of TNBC patients subjected to taxane-based neoadjuvant chemotherapy.

**Results:**

This study demonstrated that TRF2, inhibiting autophagy, is able to increase the sensitivity of TNBC cells to taxanes. The data, first obtained in *in vitro* models, were then recapitulated in preclinical mouse models and in a cohort of TNBC patients, definitively demonstrating that TRF2 over-expression enhances the efficacy of taxane-based neoadjuvant therapy in reducing tumor growth and its recurrence upon surgical intervention.

**Conclusions:**

Based on our finding it is possible to conclude that TRF2, already known for its role in promoting tumor formation and progression, might represents an Achilles’ heel for cancer. In this view, TRF2 might be exploited as a putative biomarker to predict the response of TNBC patients to taxane-based neoadjuvant chemotherapy.

**Supplementary Information:**

The online version contains supplementary material available at 10.1186/s13046-024-02998-w.

## Background

Breast Cancer (BC), with about 2.3 million of new diagnosed cases represents, to date, the most common malignancy worldwide and the leading cause of mortality for tumor in women [1]. At molecular level, BC is a highly heterogeneous disease and can be classified into multiple subtypes, which differ for their clinical management [2]. Among these, the triple negative breast cancer (TNBC) is characterized by the lacking of estrogen receptor (ER), progesterone receptor (PR) and human epidermal growth factor receptor-2 (HER2) and represents the most aggressive BC form being associated with a very poor prognosis and a high risk of mortality [3, 4]. Until now, the absence of ER, PR and HER2 expression, makes TNBC refractory to targeted and endocrine therapies, commonly used in the treatment of other BC subtypes [2]. A first step showing an improvement of outcome in TNBC patients has been the advent of immunotherapy, due to the fact that this kind of tumor is the only hot BC subtypes [5, 6]. Indeed, immunotherapy associated to chemotherapy is now the standard of care in locally advanced and in PD-L1 positive TNBC in neoadjuvant and metastatic setting, respectively [7–9].

Based on these considerations, conventional chemotherapeutic agents still represent, to date, the mainstay of strategy for the treatment of TNBC [10]. Nevertheless, the large variability observed in the drug responsiveness of patients affected by TNBC, leads to an urgent need, on one side, of developing novel and more effective therapies and, on the other side, of identifying and validating suitable markers predictive of patients' response to therapies [2].

Telomeric repeat binding factor 2 (TRF2) is one of the components of the Shelterin, a complex of six telomere-associated proteins playing a critical role in maintaining telomere integrity and genomic stability [11, 12]. During the last decade TRF2 biology has arisen renewed interest, mainly due to the discovery of novel extra-telomeric functions of the protein. Notably, a number of studies performed by our and other laboratories demonstrated that TRF2 is able to bind – similar to telomeric TTAGGG repeats – also other sequences distributed throughout the genome, the so-called interstitial telomeric sequences (ITSs) [13, 14]. In particular, these studies have demonstrated that TRF2, through its binding to the ITSs specifically located within regulatory elements (*i.e.* promoters and enhancers) of cancer-related genes and miRNAs [15–18], can promote an array of cellular processes – such as tumor angiogenesis and immune-escape – that participate, directly or indirectly, in the formation and the progression of the tumor. Consistently with these results, retrospective analyses performed on multiple patient's datasets led at defining an additional role of TRF2 as marker of aggressiveness in different tumor histotypes [17–19]. Furthermore, recent data published by our research group demonstrated that TRF2, by interacting and sequestering the cytosolic fraction of High Mobility Group B1 (HMGB1), is able to impair macro-autophagy (hereafter autophagy) [20], a highly conserved physio-pathological process playing a key role in the degradation and recycling of unnecessary and/or dysfunctional macromolecules and cellular components [21].

The role of autophagy in cancer has been largely debated, indeed, depending on the molecular characteristics of the tumor and/or the progression state of the disease, this process can either inhibit or promote the cancer formation and its aggressiveness [22]. Moreover, the autophagic process, that is constitutively active under physiological conditions, can be enhanced by several cellular stresses, included pharmacological treatments with chemo-toxic agents [21, 23, 24]. Under these conditions, autophagy can assume a detoxifying role, thus conferring drug resistance to malignant cells [25, 26].

Based on these considerations, we pointed at defining, here, if high levels of TRF2 expression might represents a sort of Achille’s heel of tumor cells, by enhancing their sensitivity toward pharmacological therapies.

To validate this hypothesis, TNBC cells – over-expressing or not TRF2 – were treated with a number of antineoplastic agents commonly used in BC therapy and drug sensitivity was evaluated both *in vitro* and in experimental *in vivo* models. These data, also confirmed in a preclinical murine model and in TNBC patients, demonstrated a direct correlation between TRF2 levels and responsiveness to taxanes, one of the most used chemotherapeutic agents in the cure of TNBC patients [27–29].

Based on these results, it is possible to conclude that TRF2 plays a role in sensitizing TNBC towards therapy and, as such, it can be considered a predictive marker of pharmacological response.

## Methods

### Cells and culture conditions

MDA-MB-231 and MDA-MB-468 human triple negative breast cancer (TNBC) cell lines were purchased from American Type Culture Collection (ATCC, Manassas, Virginia, USA) and were cultured in Dulbecco's modified Eagle's medium (DMEM; EuroClone, Italy, ECM0728L), supplemented with L-glutamine, Penicillin/Streptomycin and 10% fetal bovine serum (FBS; Thermo Fisher Scientific - Gibco, Waltham, Massachusetts, USA, 10270-106), in a CO_2_-humidified incubator at 37°C.

Stable TRF2 over-expressing and TRF2 silenced cells, and their respective control counterparts (pBabe and shSCR), were obtained as previously described [18]. For the transient expression of EGFP-LC3B, the cells were transfected for 24 hours by using JetPEI (Polyplus, Illkirch, France, 101-10N), according to the manufacturer's instructions. MDA-MB-231 LUC cells were obtained with a stable infection with lentiviral luciferase vector pRRLSIN.cPPTLuciferase (Addgene, Watertown, Massachusetts, USA).

### Reagents and treatments

Paclitaxel (Fresenius Kabi, Bad Homburg vor der Höhe, Germany), Carboplatin (Hikma, London, United Kingdom), Epirubicin (Phizer, New York, USA), Docetaxel (Accord, Milan, Italy) and Cabazitaxel (Sigma-Aldrich, St. Louis, Missouri, USA, SML2487-5MG) were used for 24 hours at the indicated final concentrations, according to the experimental settings. Chloroquine (CQ; Sigma-Aldrich, C6628) was freshly dissolved in H_2_O and used at a final concentration of 30 µM for 3 hours for WB applications and 10 µM for 24 hours for clonogenic assays.

### Fluorescence microscopy

Cells were grown on glass coverslips, treated according to the experiment, fixed in 4% formaldehyde in PBS for 10 minutes at Room Temperature (RT) and permeabilized with 0.25% Triton X-100 for 5 minutes at RT.

For autophagy experiments, cells were transiently transfected with EGFP-LC3B, as reported above, treated for 24 hours with the indicated drugs and then processed for fluorescence experiments. Autophagosome structure formation was detected by observing LC3B puncta in EGFP-LC3B expressing cells. For each experimental condition, at least 150 cells were counted; cells with more than 10 puncta were considered positive for autophagy. The results were represented as the percentage of autophagy positive cells respect EGFP-LC3B expressing cells. Nuclei were stained with DAPI. Fluorescence signals were recorded by using a Leica DMIRE2 microscope equipped with a Leica DFC 350FX camera and elaborated by Leica FW4000 deconvolution software (Leica, Wetzlar, Germany).

### Western Blot

Western blot analysis was performed as previously reported [30]. The following primary antibodies were used: Mouse mAb anti-TRF2 4A794 (Millipore, Burlington, Massachusetts, USA, 05-521); Rabbit pAb anti-LC3B (Sigma-Aldrich, L7543); Mouse mAb anti-PARP1 (BD Pharmingen, San Jose, CA, USA, Mab 551025;); Mouse mAb anti-phospho-Histone H2A.X (Ser 139; Millipore, 05-636); Mouse mAb anti-β-actin (Sigma-Aldrich, A5441). The following secondary antibodies were used: Goat anti-mouse (Biorad, Hercules, California, USA, 1706516) or anti-rabbit immunoglobulin G (IgG)-horseradish peroxidase conjugated antibodies (Biorad, 1706515).

### Clonogenic assay

MDA-MB-231 and MDA-MB-468 human TNBC cell lines were seeded in a 60mm Petri dishes, at the clonogenic density of 500 and 2000 cells/plate, respectively. The day after, the cells were treated with the indicated drugs for 24 hours. For combinatory experiments with CQ, MDA-MB-231 were pretreated with 10 mM of the autophagy inhibitor before proceeding with the previously described treatments. After 10 days, the cells were stained with 2% methylene blue in 50% ethanol and the number of colonies was counted. Surviving fractions were calculated as the ratio of absolute survival of the treated sample/absolute survival of the untreated sample, expressed as percentage.

### BrdU incorporation

The analysis of DNA and 5-bromo-2′-deoxyuridine (BrdU) contents of cells was performed as previously described [31]. Briefly, cells were pulsed with BrdU (Sigma Aldrich, B-5002) at a final concentration of 20 μM for 15 minutes, and, after a pulse (t0) or 3 hours in BrdU-free medium, the DNA was denatured. Successively, the cells were incubated with 20 μl of the mouse Mab-BrdU (Pure BD, Franklin Lakes, New Jersey, USA, 347580) for 1 hour at RT, and BrdU‐labeled cells were detected using the anti-mouse IgG (H+L), F(ab’)2 Fragment Alexa Fluor 488 Conjugate (Cell Signaling Technology, Danvers, Massachusetts, USA, 4408). Finally, the cells were counterstained with PI, acquired using FACSCelesta (BD Biosciences, San Jose, CA, USA) and analyzed with FACS Diva software (BD Biosciences).

### *In-vivo* experiments

All animal procedures were in compliance with the national and international directives (D.L. March 4, 2014, no. 26; directive 2010/63/EU of the European Parliament and European Council; Guide for the Care and Use of Laboratory Animals, U.S. National Research Council, 2011;Animal Research guidelines Reporting of In Vivo Experiments (ARRIVE) guidelines) and approved by the Italian Ministry of Health (authorization n. 607/2019-PR, released on 07-08-2019). Mice were maintained in a barrier facility on high-efficiency particulate air HEPA-filtered racks and received food and water ad libitum. CB17-SCID (CB17/Icr-Prkdcscid/IcrIcoCrl, 6 weeks old) female immunodeficient mice (Charles River Laboratories, Calco, Italy) were injected intramuscularly with 3×10^6^ of MDA-MB-231 pBabe or pTRF2 over-expressing cells. When tumors reached about 250 mm^3^, animals were randomized and treated intravenously (iv) with Paclitaxel 20 mg/Kg once a week for two weeks. Tumor volumes were measured in two dimensions using a caliper and calculated using the formula a×b^2^/2, where a and b are the long and short sizes of the tumor, respectively. Each experimental group included five mice. At the end of the experiment mice were sacrificed for ethical reasons and tumors were excised for IHC analysis.

To perform orthotopic experiment, NOD.Cg-PrkdcSCID IL-2R null (NSG, 6 weeks old) female immunodeficient mice (Charles River Laboratories) were orthotopically injected in the mouse mammary gland with 1x10^6^ MDA-MB-231 LUC pBabe or pTRF2 cells using an insulin syringe with a 27-gauge needle. Mice were anesthetized with a combination of tiletamine–zolazepam (Telazol, Virbac, Carros, France) and xylazine (xylazine/Rompun, Bayer, Leverkusen, North Rhine-Westphalia, Germany) given intramuscularly at 2 mg/kg, and a small incision of less than 3 mm was made externally and caudally to the nipple. With the aid of micro-dissecting forceps, the ventral most part of the fat pad was gently pulled out and exposed through the small incision [32]. Successful injection was confirmed by the swelling of the tissue. The small incision was sealed using absorbable suture (PolySorbTM 5-0). Finally, mice were medicated with an orally administration of 0.5 mg/Kg of Metacam (meloxicam) to control post-operative pain and inflammation. When the tumors were palpable (day 10 after cells injection), mice were randomized and treated intravenously (iv) with Paclitaxel 20 mg/Kg once a week for two weeks. Real time tumor growth was monitored weekly using the IVIS Lumina II CCD camera system (PerkinElmer, Shelton, Connecticut, USA) by intraperitoneally injection with 150 mg/Kg D-Luciferin (PerkinElmer). Bioluminescence signals were determined by the number of photons and were acquired and analyzed using the Living image software version 4.3 (PerkinElmer). Fifteen days post treatment, primary tumors were surgically resected and mice were monitored by IVIS imaging for metastases appearance.

Each experimental group included 8 mice. The student’s t-test (unpaired, two-tailed) was used for single pair-wise comparisons. Differences were considered statistically significant when P < 0.05. Survival curves of mice were processed using the Kaplan-Meier method, and statistical significance was assessed by log-rank test. Data were plotted using GraphPad Prism Software 7 (GraphPad Software, Boston, MA, USA).

### Immunohistochemistry (IHC)

Immunohistochemistry (IHC) analyses were performed as in [Iachettini 2023]. After sectioning and processing, the tissue sections were immunostained for 1 hours at RT with anti-TERF2/TRF2 rabbit polyclonal (1:500), anti-LC3B rabbit monoclonal (AbCam, Cambridge, United Kingdom, EPR21234, 1:100), anti-p62 SQSTM1 mouse monoclonal (D-3) (Santa Cruz Biotechnology, Dallas, Texas, USA, sc-28359, 1:300), anti-Ki67 mouse monoclonal (Agilent Dako, Santa Clara, CA, USA, MIB-1, 1:100), and anti-Cleaved Caspase-3 rabbit polyclonal (Cell Signaling, #9661, 1:100) antibodies and then were covered for 30 minutes at RT with Dako EnVision™ FLEX /HRP (EnVision™ FLEX; Agilent, Santa Clara, CA, USA, K8023). The signal was developed by using DAB detection kit (Agilent Dako, GV825), then sections were counterstained with Mayer’s Hematoxylin (Agilent Dako, S3309). Finally, slides were washed, dehydrated and mounted with Eukitt (Sigma-Aldrich, 03989). Immunostaining results were recorded as percentage of positive cells or immunoreactive score (IRS, staining intensity per percentage of positive cells).

### Patients

Data were retrieved from 49 TNBC patients who underwent neoadjuvant chemotherapy including anthracycline and taxanes (+/- Carboplatin) until to 8 months at the IRCCS Regina Elena National Cancer Institute and IRCCS Fondazione Policlinico Universitario Agostino Gemelli, from July 2009 to July 2020.

For this retrospective analysis, patients were considered eligible if their tissue from pre-surgical biopsy was available and if the neoadjuvant treatment was completed. Clinical and bio-pathological information was retrieved from medical records. Pathological complete response (pCR) to neoadjuvant treatment was defined as absence of residual of invasive tumor in both breast and axilla after completion of neoadjuvant therapy. This study was conducted in accordance with the Declaration of Helsinki and was approved by the Ethic Committee of Regina Elena National Cancer Institute as coordinating center (RS N° 09/23), and other participating centers.

TRF2 labeling was performed on tissue sections as reported in the previous paragraph and its expression was quantified according to immunoreactive score (IRS) as reported in Table 1.

**Table 1:** IRS classification

**Percentage of positive cells X Intensity of Staining = Score (0-12) IRS - classification**			
0 = no positive cells	0 = no color reaction	0 - 1 = negative	0 = negative
1 = < 10% positive cells	1 = mild reaction	2 - 3 = mild	1 = positive, weak expression
2 = 10-50% positive cells	2 = moderate reaction	4 - 8 = moderate	2 = positive, intermediate expression
3 = 51-80% positive cells	3 = intense reaction	9 - 12 = strongly positive	3 = positive, strong expression
4 = >80% positive cells			

### Statistical Analysis

Experiments were replicated three times and the data were expressed as means ± standard deviation (SD). Each experimental group included 8 mice. The student’s t-test (unpaired, two-tailed) was used for single pair-wise comparisons. Differences were considered statistically significant when *P* < 0.05. Survival curves of mice were processed using the Kaplan-Meier method, and statistical significance was assessed by log-rank test. Data were plotted using GraphPad Prism Software 7 (GraphPad Software, Boston, MA, USA). Differences were considered statistically significant for *p < 0.05; **p< 0.01; ***p < 0.001; ****p<0.0001. For patients’ sample, descriptive statistics were calculated for all variables of interest. The obtained distribution was compared by neoadjuvant response status with the Mann-Whitney non parametric test. Correlation between continuous variables was tested by Spearman’s non parametric correlation coefficient. Potential differences between TRF2 values pre and post neoadjuvant therapy were tested with the Wilcoxon non parametric test. Kaplain-Meier method was used to carry out event free survival (EFS) analysis. EFS was calculated as the time between the first cycle of neoadjuvant chemotherapy until evidence of disease progression or death due to any cause. The log-rank test was used to detect potential differences between subgroups. A *p*-value *(p*)<0.05 was considered statistically significant. The analyses were carried out with IBM SPSS v.29.0.

## Results

### TRF2 enhances chemo-sensitivity of TNBC cells by inhibiting autophagy

Autophagy is a finely tuned process playing a key role in the maintenance of cellular homeostasis. As such, the autophagic process has been found to be activated in response to a wide variety of stresses, so assuming a pro-survival role. In particular, activation of autophagy in response to the exposure to cytotoxic agents, included antineoplastic drugs, has been demonstrated to exert a detoxifying role in cancer cells [25, 26].

**Fig. 1:**
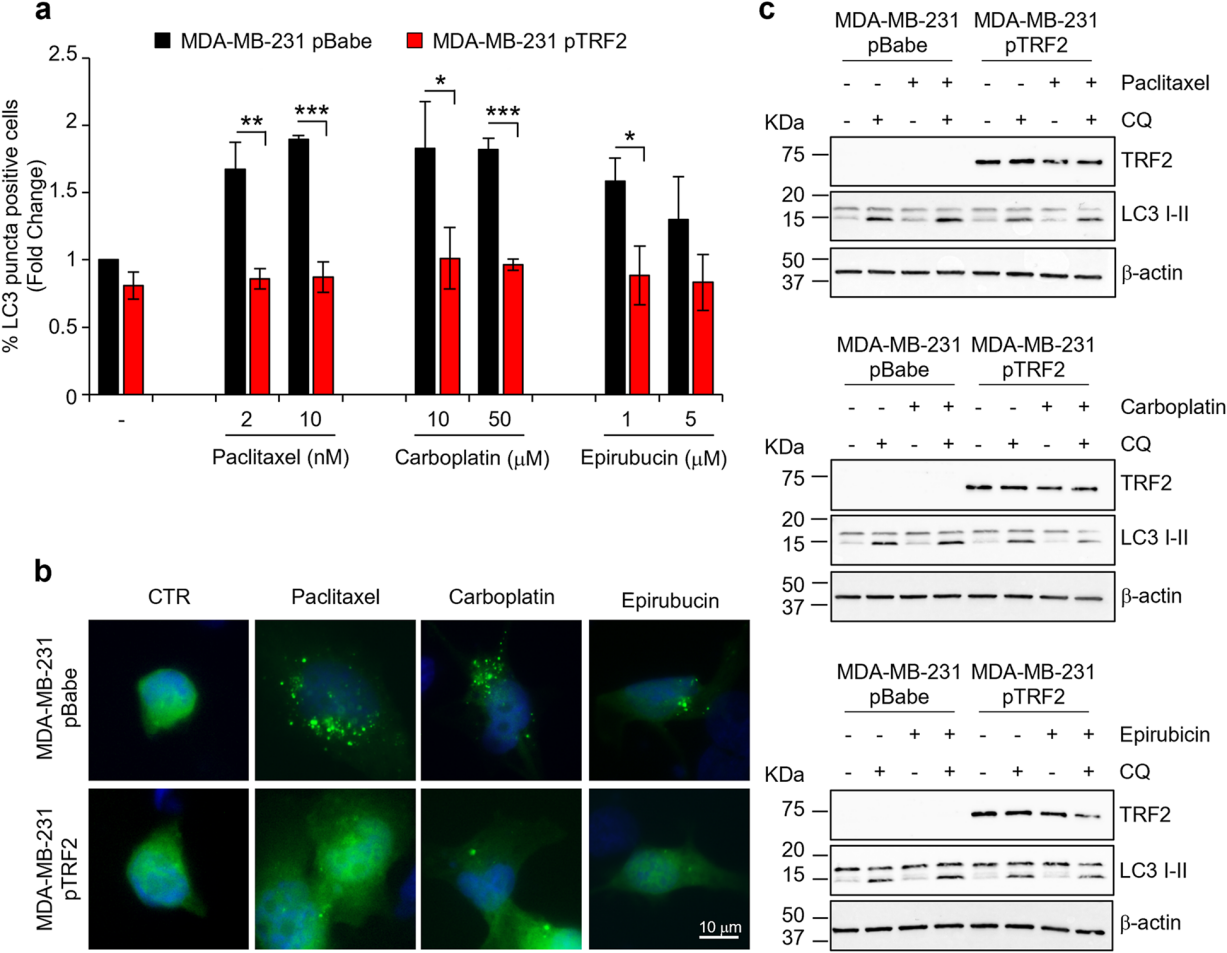
TRF2 impairs autophagy induced by chemo-therapeutic agents in TNBC. **a** Human triple negative breast cancer (TNBC) cell lines MDA-MB-231, over-expressing (pTRF2) or not (pBabe) TRF2 were transfected with EGFP-LC3 for 24 hours and treated with Paclitaxel, Carboplatin and Epirubicin for 24 hours at the indicated doses. The autophagic process was evaluated by the quantitative analysis of punctate vesicular structures by fluorescence experiments. Histogram represented the percentage of LC3 puncta positive cells on total GFP positive cells. **b** Representative images of the experiment described in **a** were acquired by deconvolution microscopy (63X magnification). Blue: the nuclei stained with DAPI. Green: EGFP-LC3B. **c** Western blot analysis of LC3 I-II protein levels in MDA-MB-231 pBabe or pTRF2 treated with the indicated drugs in combination or not with 30 µM of the autophagy inhibitor Chloroquine (CQ) for 3 hours. TRF2 protein levels were monitored to check cell lines and b-actin as loading internal control. The histograms represent the mean values ± S.D. of three independent experiments; *p<0.05, **p<0.01, ***p <0.001.

Here, based on our recent data showing a role of TRF2 in inhibiting autophagy [20], we pointed at evaluating, on one hand, the capability of a panel of chemotherapeutic agents to promote autophagic response in TNBC cells and, on the other hand, if the over-expression of TRF2 is able to impair the eventual pro-autophagic activity of these drugs. To this aim, a well-established TNBC model, MDA-MB-231 cells, over-expressing (pBabe-TRF2) or not (pBabe-empty) TRF2 (Fig. S1a), underwent to treatment with Paclitaxel, Epirubicin and Carboplatin – three drugs representative of main classes of cytotoxic agents (taxanes, platins and anthracyclines) used for the treatment of BC – and the entity of autophagy was evaluated (Fig. 1). For these experiments, the cells were transfected with EGFP-tagged LC3B (EGFP-LC3B) and the percentage of cells with EGFP-LC3B foci (LC3 puncta-positive cells) was quantified. Interestingly, the fluorescence analyses – then biochemically confirmed by Western Blot (WB) analyses – demonstrated that the all the tested drugs are able to promote a potent autophagic response that is impaired by TRF2 over-expression (Fig. 1a-c).

Among the tested drugs, taxanes, a family of natural molecules promoting their cytotoxic activity by affecting microtubule assembling dynamics, represent the gold standard for neoadjuvant treatment of TNBC patients [27–29, 33]. Based on this, we decided to mainly focus on this class of molecules, by extending our analyses also to other two taxanes: docetaxel and cabazitaxel [34]. Of note, the experiments performed in the MDA-MB-231 (Fig. S1b, c), and then confirmed in MDA-MB-468 (Fig. S 1d-f), evidenced that autophagy is a generalizable response of TNBC cells to taxane-based treatment and that TRF2 is able to inhibit, significatively, the capability of this drugs’ family to promote autophagy.

**Fig. 2:**
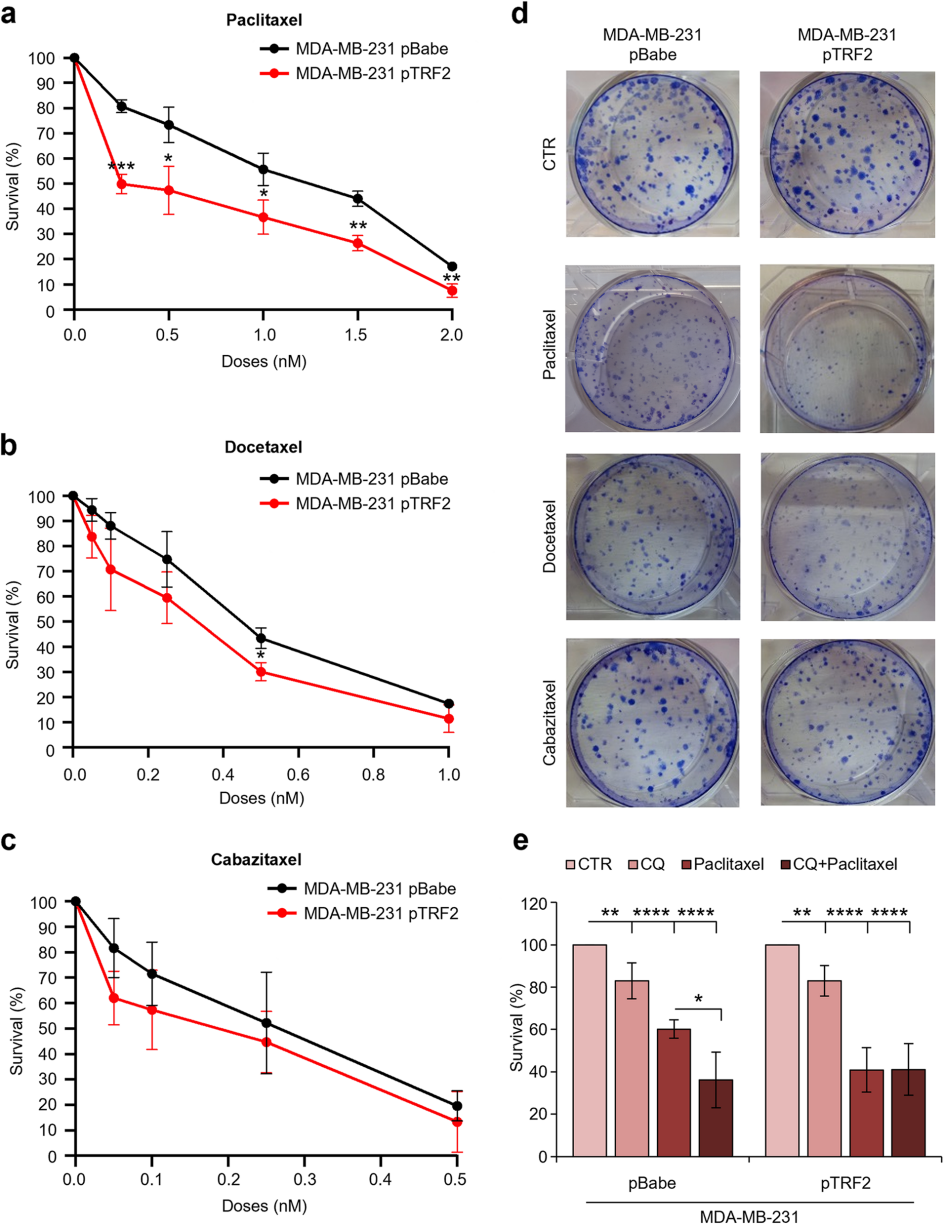
TRF2 enhances TNBC cell sensitivity to taxanes. **a-c** Cell survival evaluation by clonogenic assay in human triple negative breast cancer (TNBC) cell lines, MDA-MB-231, over-expressing (pTRF2) or not (pBabe) TRF2 and treated with three taxanes (Paclitaxel **a**, Docetaxel **b** and Cabazitaxel **c**) at the indicated doses for 24 hours. **d** Representative images of the clonogenic assays described in **a-c**. **e** Clonogenic assay performed in TNBC cell lines MDA-MB-231 pBabe or pTRF2 pretreated with Chloroquine (CQ) 10 mM for 24 hours before the administration of Paclitaxel 1 nM for 24 hours. The graphs represent the mean values ± S.D. of at least three independent experiments. *p<0.05, **p<0.01, ***p <0.001, ****p<0.0001.

Next we pointed at evaluating whether the over-expression of TRF2, inhibiting autophagy, was able to affect the drug sensitivity of tumor cells. To this aim, TNBC cells were treated or not with the different taxanes and clonogenic activity was evaluated. Interestingly, results of colony-formation assays (Fig. 2a-d and Fig. S2a), paralleled by WB and fluorescence-activated cell sorter (FACS) analyses (Fig. S2b, c), demonstrated that increased levels of the TRF2 enhance the susceptibility of TNBC cells towards taxane-based therapies. In agreement with these results, our data demonstrated that Paclitaxel impairs TRF2 levels (Fig. S2b), suggesting that over-expressing cells – as confirmed by cell proliferation inhibition and cell death enhancement (Fig. S2b, c) – are more sensitive to treatment. For completeness, sensitivity to taxanes was evaluated also in MDA-MB-231 cells silenced for TRF2. As evidenced by colony-formation assay (Fig. S3a), impairment of TRF2 expression reduced cell sensitivity to Paclitaxel, confirming and reinforcing the key role of the protein in affecting TNBC response to therapy.

Finally, to demonstrate that the increased drug-sensitivity of cells over-expressing TRF2 is effectively due to the capability of the protein to impair autophagy, MDA-MB-231 cells were subjected to treatment with a specific inhibitor of autophagic process, Cloroquine (CQ), and the activity of Paclitaxel was evaluated. Interestingly the results of colony formation assays (Fig. 2e), demonstrated that the exposure of the cells to CQ sensitizes TNBC cells to taxane treatment, fully recapitulating the effect of TRF2 over-expression. Moreover, CQ did not show additive effect to Paclitaxel treatment in TRF2 over-expressing cells, confirming that capability of TRF2 to enhance cell sensitivity to taxanes goes essentially through autophagy inhibition. Given the well-known role played by TRF2 in DNA damage response, the levels of phosphorylated histone H2AX (γH2AX), a well-defined marker of DNA damage, were evaluated in MDA-MB-231 cells over-expressing TRF2 (pTRF2), and in their control counterpart (pBabe), in absence and in presence of Paclitaxel. Despite a slight increase of DNA damage upon taxane exposure, results of WB analyses (Fig. S3b) evidenced no significative differences related to TRF2 modulation, indicating that TRF2-dependent DNA damage does not contribute, under the evaluated conditions, at promoting taxane-sensitivity.

### TRF2 enhances sensitivity of TNBC to taxane-based therapy *in vivo*

**Fig. 3:**
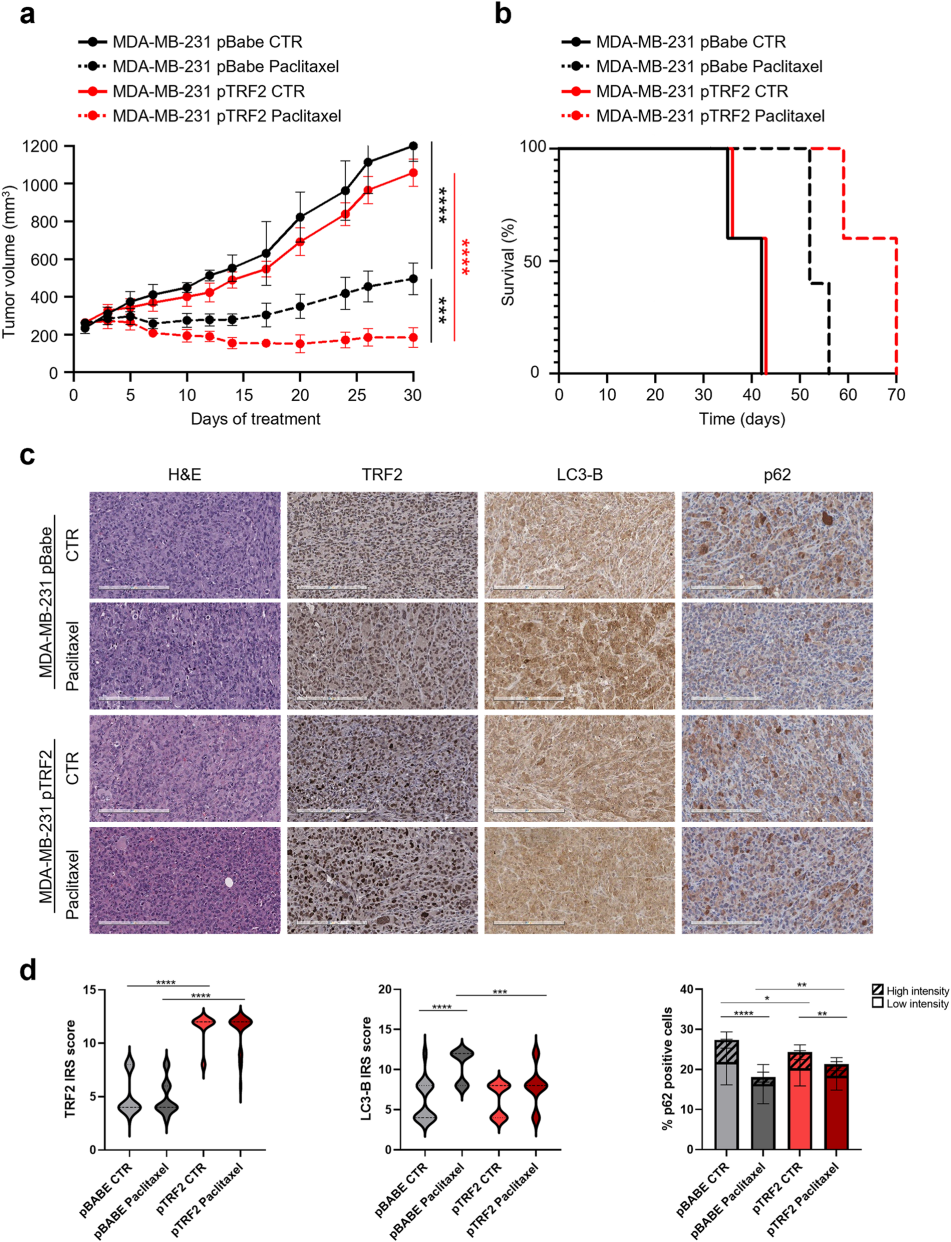
Paclitaxel efficacy in xenograft TNBC tumors is enhanced by TRF2 over-expression. **a** CB17-SCID female immunodeficient mice were injected intramuscularly with 3x10^6^ MDA-MB-231 pBabe and pTRF2 cells. When tumors reached 250 mm^3^ mice were randomised and treated with Paclitaxel *iv* at 20 mg/Kg once a week for two weeks. Tumor volumes were measured in two dimensions using a caliper and calculated by the formula a× b^2^/2, where “a” and “b” are the long and short sizes of the tumor, respectively. Each experimental group included five mice. Error bars represent S.D. and *p*-values were calculated at day 30 using an unpaired two-tailed t-test; ***p<0.001, ****p<0.00001. b Kaplan-Meier survival curves for mice treated as in **a**. Statistical significance was assessed by Log-rank test (****p<0.0001). **c** Histological and Immunohistochemical analysis of primary tumor established from MDA-MB-231 pBabe and pTRF2 treated with Paclitaxel. Representative images of immunostained sections. Scale bar: 200 µm. **d** Quantification of TRF2 and LC-3B (expressed as ImmunoReactive Score, IRS) and SQSTM1/p62 expression (indicated as percentage of SQSTM1/p62 positive cells). Thirty fields for condition were analyzed. *p<0.05, **p<0.01, ***p <0.001, ****p<0.0001.

Based on the obtained results, we moved at confirming our findings in advanced *in vivo* BC models. In detail, we first evaluated the existence of a positive correlation between TRF2 expression levels and tumor sensitivity to Paclitaxel in xenograft mice. To this aim, MDA-MB-231 cells, over-expressing (pTRF2) or not (pBabe) TRF2, were intramuscularly injected into immunocompromised SCID mice and, upon tumor establishment, the animals underwent to treatment with the drug. As expected, intravenous injection of Paclitaxel (20 mg/kg once a week for two weeks) promoted a robust impairment of tumor growth in all the animals and, consistently with the *in vitro* results, over-expression of TRF2 enhanced significatively tumor sensitivity to the drug (Fig. 3a).

Moreover, while Paclitaxel induced stabilization of the disease in 3/5 pBabe mice, over-expression of TRF2 exacerbated the effect of the drug by promoting the disease stabilization in 4/5 animals and a complete response in 1/5 treated xenografts (Table 2). In line with these results, we observed an increase of animal survival of about 30% in treated TRF2 xenografts over the pBabe counterpart (Fig. 3b and Table 2). In line with these results, *ex-vivo* immunohistochemical (IHC) analyses evidenced that Paclitaxel promotes a significant reduction of proliferation marker, Ki67, associated with a slight increase of Caspase-3, a common marker of apoptosis (Fig. S4). Furthermore, in accordance with the *in vitro* results, over-expression of TRF2 inhibits the autophagic response induced by the treatment with Paclitaxel. Indeed, tumors over-expressing TRF2 showed reduced levels of LC3, associated with an accumulation of p62 SQSTM1, upon drug exposure (Fig. 3c-d), confirming that reduction of tumor volume observed in response to treatment is largely attributableto a robust impairment of TRF2-dependent autophagy activation.

**Table 2:** Antitumor efficacy of Paclitxel against MDA-MB-431 pBabe and pTRF2 xenografts

**Treatment groups**	**Tumor volume inhibition**^**a**^	**Stable disease**^**b**^	**Complete response**^**c**^	**ILS**^**d**^** (%)**	**Body weight loss**^**e**^** (%)**
MDA-MB-231 pBabe+PACLI	57	3/5	0/5	33	2
MDA-MB-231 pTRF2+PACLI	78	4/	1/5	63	2

Following transplantation, tumors were allowed grow for 7 days to about 250 mm^3^ before initiation of treatment (day 1). Mice were treated with PACLI *iv* at 20 mg/kg at day 1 and 8

^a^Tumor volume inhibition was calculated as the nadir of the effect by the following formula: (tumor volume treated mice/tumor volume untreated mice-1) x 100

^b^Stable disease was defined as the maintaining of the same tumor volume, after the initiation of treatment, for at least three weeks

^c^Complete response was defined as disappearance of tumor for at least two weeks

^d^Increase of life survival. The animals were euthanized for ethical reasons when tumors reached a mean of 1.2 cm^3^ in volume and the time of euthanization was recorded as the time of death

^e^The percentage of body weight loss was calculated after the initiation of treatment

**Fig. 4:**
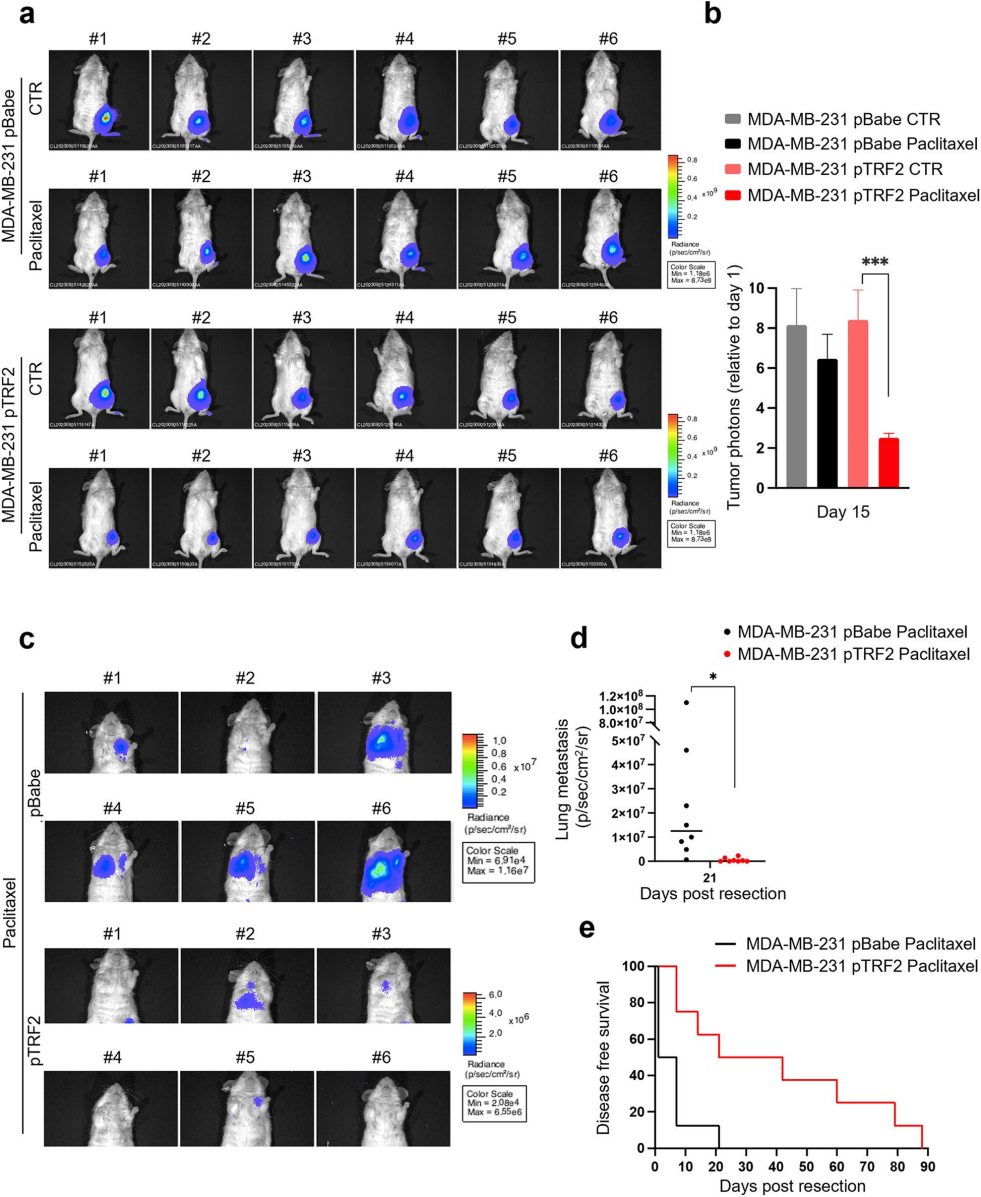
TRF2 over-expression impairs tumor growth and dissemination in advanced orthotopic TNBC models. NSG female immunodeficient mice were orthotopically injected in the mouse mammary gland with 1x10^6^ MDA-MB-231-LUC pBabe and pTRF2 cells. When tumors were palpable (day 10 after cells injection), mice were randomized and treated intravenously with vehicle, or Paclitaxel *iv* at 20 μg/mouse once a week for two weeks. At the end of the treatment (15 days post treatment) mice were analyzed by the IVIS imaging system 200 series and bioluminescence signals were determined by the number of photons analyzed using the Living image software version 4.3 (PerkinElmer). **a** Representative pictures of six mice. **b** Quantitative analyses of bioluminescence signals were shown. Luminescent signals, relative to photons at the beginning of treatment, are expressed as mean of total flux of photons/sec/cm^2^/steradian (p/s/cm^2^/sr). Each experimental group included *n*=8 mice. Error bars represent SEM. *P* values were calculated using an unpaired two-tailed t-test, ***p<0.001. **c,d** Fifteen days post treatment, primary tumors were surgically resected and mice treated with Paclitaxel were monitored for lung metastasis appearance by IVIS imaging system 200 series. Representative pictures of six mice, at day 21 post tumor resection **c** and quantitative analysis of lung metastasis photons **d** were shown. *P* values were calculated using an unpaired two-tailed t-test, *p<0.05. **e** Kaplan-Meier disease free survival curve of mice as in **C**, with each experimental group included *n*=8 mice. Statistical significance was assessed by Log-rank test (**p=0.0046)

Finally, to mimic the clinical protocols adopted for treatment of TNBC patients, we extended our findings to an advanced orthotopic model in which we evaluated tumor progression upon neoadjuvant chemotherapy, followed by surgical resection of the tumor. To this aim, luminescent MDA-MB-231 cells, over-expressing (pTRF2) or not (pBabe) TRF2, were inoculated in the mammary fat pad of immune-compromised mice and the tumor growth was monitored by IVIS imaging. Similar to results obtained in the xenograft models, tumors over-expressing TRF2 resulted dramatically more sensitive to treatment with Paclitaxel than their control counterpart (Fig. 4a, b), evidencing a growth inhibition of about the 70% over untreated tumors. Upon completion of treatment (day 15), residual primary tumors were surgically removed (Fig. S5) and the formation of spontaneous metastases was monitored along the time, by bioluminescence analysis (Fig. 4c, d). Interestingly, while all the treated mice from pBabe group developed distal metastases at day 21 post-resection, only 4/8 animals from the TRF2 over-expressing group showed lung metastases at the same day. For completeness, this latter group was monitored up to day 88 when all mice were positive for metastases (Fig. 4e), underlying that high levels of TRF2 correlate with a substantial delay in tumor progression following neoadjuvant therapy with taxanes.

### TRF2 is a predictive marker of response to taxane-based therapy in TNBC patients

In the last part of this work we pointed at defining the clinical relevance of our findings. To address this objective, we performed a retrospective study on 49 women with TNBC who received taxane-based neoadjuvant chemotherapy at the IRCCS Regina Elena National Cancer Institute and IRCCS Fondazione Policlinico Universitario Agostino Gemelli in a period ranging from July 2009 to July 2020.

As specified in Table 3, patients included in the study had a median age of 49 years at diagnosis and they were exposed to neoadjuvant regimen for a median of 5 months (range: 2-8 months). Notably, among 49 recruited patients, one discontinued therapy early due to disease progression. A total of 18 patients (36.7%) had a pathological complete response, since there was no residual invasive tumor at surgery after neoadjuvant therapy.

**Table 3:** Patients’ characteristics

**Parameter**	**N (%)**
**N° of patients **	**49 **
**Age at diagnosis (years) **	
Median (min-max)	49 (29-76)
**Menopausal status**	24 (49.0)
**HER2 status**	
0	41 (83.7)
1+	6 (12.2)
2+SISH^a^/FISH^b^ not amplified	2 (4.1)
**Chemotherapy Regimens**	
Antracycline+Taxanes	33 (67.3)
Antracylcine+Taxanes+Carboplatinum	16 (32.7)
**TRF2 IRS **^**c**^** categories**	
TRF2Low	12 (24.5)
TRF2High	37 (75.5)
**Ki-67 pre neo-adjuvant**	
Median (min-max) in %	60 (12-95)
**pCR**^d^	
Yes	18 (36.7)
No	31 (63.3)
**Median duration of chemotherapy(months)**	5 (2*-8)
Progressive patients	12 (25.0)
No progressive patients	36 (75.0)
**Overall Survival**	
Death	8 (16.7)
Alive	40 (83.3)

^a^*SISH* Silver in situ hybridization

^b^*FISH* Fluorescence in situ hybridization

^c^*IRS* Immunoreactive score

^d^*pCR* Pathological complete response

**Fig. 5:**
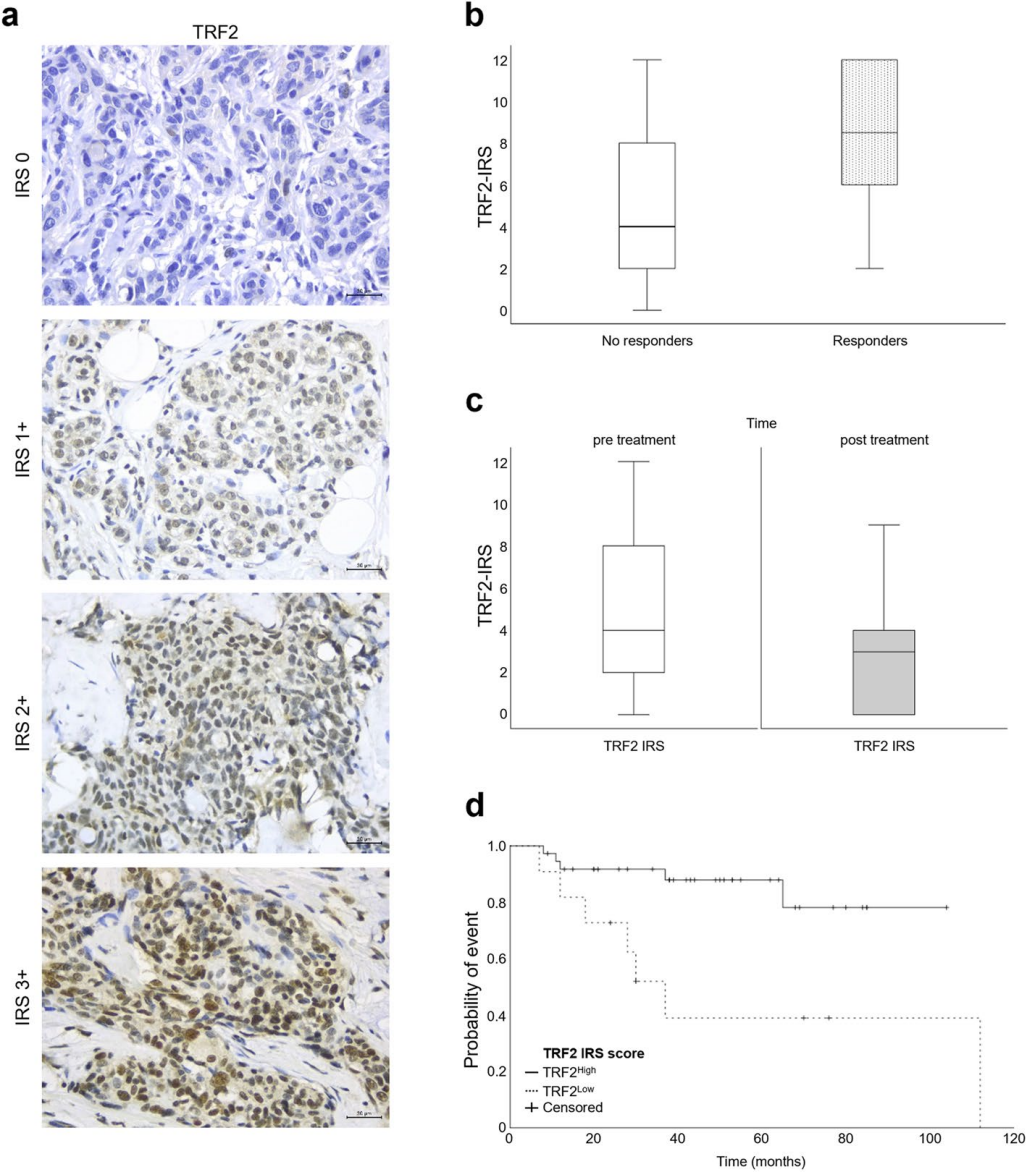
High TRF2 levels in TNBC patients correlate with a better response to neoadjuvant taxane-based therapy. **a** Representative IHC images of TRF2 score in tumor specimens retrieved from TNBC patients before neoadjuvant therapy. Scale bar: 30 µm. **b** TRF2-IRS distribution by patients’ response to taxane-based neoadjuvant therapy. Responders showed TRF2 IRS values significantly higher than no responders (Mann-Whitney test, *p*=0.010). **c** Analysis of TRF2 IRS value pre- and post- neoadjuvant taxane-based therapy. Post treatment TRF2-IRS values were significantly lower than pre-treatment (Wilcoxon test, *p*=0.005). **d** Kaplan-Meier EFS curves of TNBC patients subjected to neoadjuvant taxane-based therapy. Patients with TRF2 IRS^High^ had a longer time without disease recurrence than those with TRF2^Low^ (TRF2 IRS^High^ median time not estimable vs TRF2^Low^ median time 37 months 95%CI [4.7- 49.3], Log-rank test, *p*=0.005).

In line with the aim of the work, tumor specimens retrieved from patients before neoadjuvant therapy were subjected to IHC analysis to evaluate the levels of TRF2 expression (Fig. 5a). According to the immunoreactive score (IRS), calculated as TRF2 staining intensity per percentage of positive cells (Table 1), 12 patients (24.5%) were categorized as TRF2^Low^ (IRS: 0-1) while the remaining 37 (75.5%) were TRF2^High^ (IRS: 2-3). In order to define the existence of a correlation between TRF2 expression levels and the activity of neoadjuvant therapy in terms of response, TRF2-IRS distribution was compared between the group of responder and no responder patients. Notably, this analysis evidenced higher median TRF2 values in patients who achieved a pCR (Fig. 5b), suggesting the use of TRF2 as a reliable marker to predict the response of TNBC patients to taxane-based neoadjuvant chemotherapy.

Interestingly, additional analysis of TRF2-IRS values performed in 27 out of 31 no responder patients (depending on material availability) revealed a significative decrease of TRF2 levels after neoadjuvant therapy (Fig. 5c, Wilcoxon test, *p*=0.005). This result, in accordance with the *in vitro* finding (Fig. S2b), would support the hypothesis that fraction of tumor cells with higher TRF2 levels is more sensitive to taxane-based neoadjuvant therapy.

Finally, we investigated whether TRF2 could have a role in regulating the progression of disease following neoadjuvant therapy and surgical resection of residual tumor. Event free survival data were available for 48 patients, with a median follow-up of 43 months (range 8-112). In accordance with previous results, Kaplan-Meier curves showed that TRF2^High^ patients had a longer time to disease recurrence than the TRF2^Low^ ones (Fig. 5d, log-rank test *p*=0.005). In detail, the proportion of patients without disease recurrence was 92% for the TRF2^High^ and 73% for the TRF2^Low^ at 24 months and raised to 88% and 39%, respectively, at 60 months (Fig. 5d).

## Discussion

Despite the relevant oncological progresses of the last few years, management of TNBC still represents an unmet medical need. Indeed, lacking ER, PR and HER2 expression, patients affected by this kind of tumor do not benefit of the therapeutic regimens (*i.e.* endocrine and target therapies) effective against other BC subtypes [2]. Indeed, the standard protocols for treatment of TNBC patients are based on administration of neoadjuvant chemotherapy, recently associated to immunotherapy, followed by surgical resection [35]. In particular, taxanes – a family of well-tolerated chemotherapeutic agents able to inhibit cell cycle progression through microtubules’ stabilization – have been demonstrated to be particularly effective in treatment of TNBC and, as such, they are indicated by the most recent clinical guidelines as the gold therapeutic standard for treatment of TNBC patients [27–29, 33]. Recent studies in TNBC patients treated with neoadjuvant chemo-immunotherapy have shown clinically significant improvements in outcome [36, 37]. In particular, the phase 3 KEYNOTE-522 study showed that pembrolizumab, administered in combination with neoadjuvant chemotherapy including anthracyclines and taxane, and then continued as adjuvant monotherapy, resulted in a statistically significant and clinically meaningful improvement both in terms of pCR (58.7% vs 40.0% (95%) and 36-month EFS rate (91.2% vs 77.2%), regardless of PD-L1 status [7, 8]. Consequently, pembrolizumab was recently approved in locally advanced TNBC neoadjuvant setting in combination with anthracycline, taxane and carboplatin-based therapy. Certainly, an improved understanding of tumor biology and mechanisms of action/resistance to taxanes will lead to significant advances in results, but – despite the encouraging clinical data – the variability of response to taxanes raised the urgent need of finding predictive markers to identify patients that would most benefit of this kind of therapy [38].

As known, cancer cells are able to counteract cytotoxic stresses by activating an array of defensive mechanisms. Autophagy, a degradative process physiologically relevant for the maintenance of cell homeostasis [21], has been found to exert a protective role in cancer, mainly through its capability of promoting a detoxifying activity towards a number of antineoplastic drugs [25, 26]. In this view, recent works demonstrated that the treatment with taxanes enhances autophagy in BC cells [39–41] and that pharmacological inhibition of this autophagic response exacerbates the cytotoxic activity of this family of chemotherapeutic agents [42–45].

Based on these considerations and supported by our recently published data showing a role of the telomeric protein TRF2 in impairing autophagy [20], we pointed at defining, here, the existence of a direct correlation between the expression level of TRF2 and the efficacy of taxanes in TNBC patients. As detailed in the results, the obtained *in vitro* data confirmed, first, the capability of different taxanes (*i.e.* paclitaxel, docetaxel and carbazitaxel) to promote an autophagic response in TNBC cell lines and highlighted – at the same time – the capability of TRF2 to impair drug-induced autophagy, leading to a dramatic reduction of cancer cells proliferation. In addition, the reduction of TRF2 levels observed in TNBC cells subjected to taxane-based therapy would suggest, in accordance with our initial hypothesis, that TNBC cells are as more sensitive to this family of chemotherapeutic agents as higher are the levels of TRF2.

In agreement with these data, analyses performed in preclinical animal models confirmed that Paclitaxel is markedly more effective in tumors originated from TRF2 over-expressing cells than in their control counterpart. In particular, *ex-vivo* IHC analyses performed on the sections of excised tumors demonstrated that high TRF2 levels impair the autophagic response induced by Paclitaxel.

Even if we cannot exclude that other mechanisms might play a role in conferring sensitivity to taxane-based therapy, the obtained data strongly suggest the existence of a correlation between autophagy and tumor proliferation in animals subjected to chemotherapy. In addition, TRF2 over-expressing tumors treated with Paclitaxel in a neoadjuvant setting also showed a reduction in the appearance of metastases upon surgical intervention. Interestingly, being TRF2 expression correlated with the aggressive phenotype of different tumor histotypes, included TNBC, it is reasonable to suppose that reduced tumor dissemination following the treatment with taxanes is attributable to selective killing of the fraction of tumor cells expressing high TRF2 levels.

Finally, we pointed at evaluating the clinical relevance of our findings by performing a retrospective analysis on a dataset of TNBC patients who received taxane-based neoadjuvant therapy. Based on our results, it is possible to conclude that TRF2 plays a role in sensitizing TNBC towards therapy and, as such, it can be considered a predictive marker of pharmacological response and a prognostic biomarker in TNBC patients. In this view, being TRF2 levels easily quantifiable through not invasive methodologies, it is reasonable to auspicate that the analysis of this protein would routinely enter in the clinical practice. An important challenge will be to investigate in a prospective investigation the correlation between TFR2 expression with the new neoadjuvant regimens including immunotherapy.

## Conclusions

The obtained results well support the idea that TRF2, despite promoting cancer formation and progression, is able to increase the sensitivity of tumor cells to taxane-based chemotherapy, representing a sort of Achilles' heel for TNBC cells. Based on this, TRF2, might be considered a good candidate for further studies aimed at validating its dual role both as a molecular target for the development of new therapies and as a marker to predict the response of patients to taxane-based chemotherapy.

### Supplementary Information

**Supplementary Material 1.**

## Data Availability

The data that support the findings of this study are available from the corresponding authors upon reasonable request.

## Abbreviations

ATCC: American Type Culture Collection
BC: Breast Cancer
BrdU: 5-Bromo-2′-deoxyuridine
CQ: Chloroquine
DAB: 3,3′-Diaminobenzidine
DMEM: Dulbecco's modified Eagle's medium
ER: Estrogen receptor
FACS: Fluorescence-activated cell sorter
FBS: Fetal bovine serum
HER2: Human epidermal growth factor receptor-2
HMGB1: High Mobility Group B1
IHC: Immunohistochemistry
IRS: Immunoractive score
ITS: Interstitial telomeric sequence
IV: Intravenous
LC3: Light chain 3
NSG: NOD.Cg-PrkdcSCID IL-2R null
PBS: Phosphate Buffered Saline
PFS: Progression free survival
PR: Progesterone receptor
RT: Room Temperature
SCID: Severe combined immunodeficiency
SD: Standard deviation
TNBC: Triple negative breast cancer
TRF2: Telomeric repeat-binding factor 2
WB: Western Blot
