# Supplementary material for: TRF2 as novel marker of tumor response to taxane-based therapy: from mechanistic insight to clinical implication
Source: J Exp Clin Cancer Res. 2024 Mar 9;43:75. doi: 10.1186/s13046-024-02998-w (PMC10924347; doi:10.1186/s13046-024-02998-w)
Supplement: Supplementary file 1 — Supplementary Material 1. [file 13046_2024_2998_MOESM1_ESM.pdf]

SUPPLEMENTARY FIGURES

TRF2 as a novel marker of tumor response to taxane-based therapy: from mechanistic insight to clinical implications.

Sara Iachettini, Irene Terrenato, Manuela Porru, Serena Di Vito, Angela Rizzo, Carmen D'Angelo, Eleonora Petti, Roberto Dinami, Carmen Maresca, Anna Di Benedetto, Aldo Palange, Antonino Mulè, Angela Santoro, Antonella Palazzo, Antonella Stoppacciaro, Patrizia Vici, Lorena Filomeno, Francesca Sofia Di Lisa, Teresa Arcuri, Eriseld Krasniqi, Alessandra Fabi, Annamaria Biroccio, Pasquale Zizza.

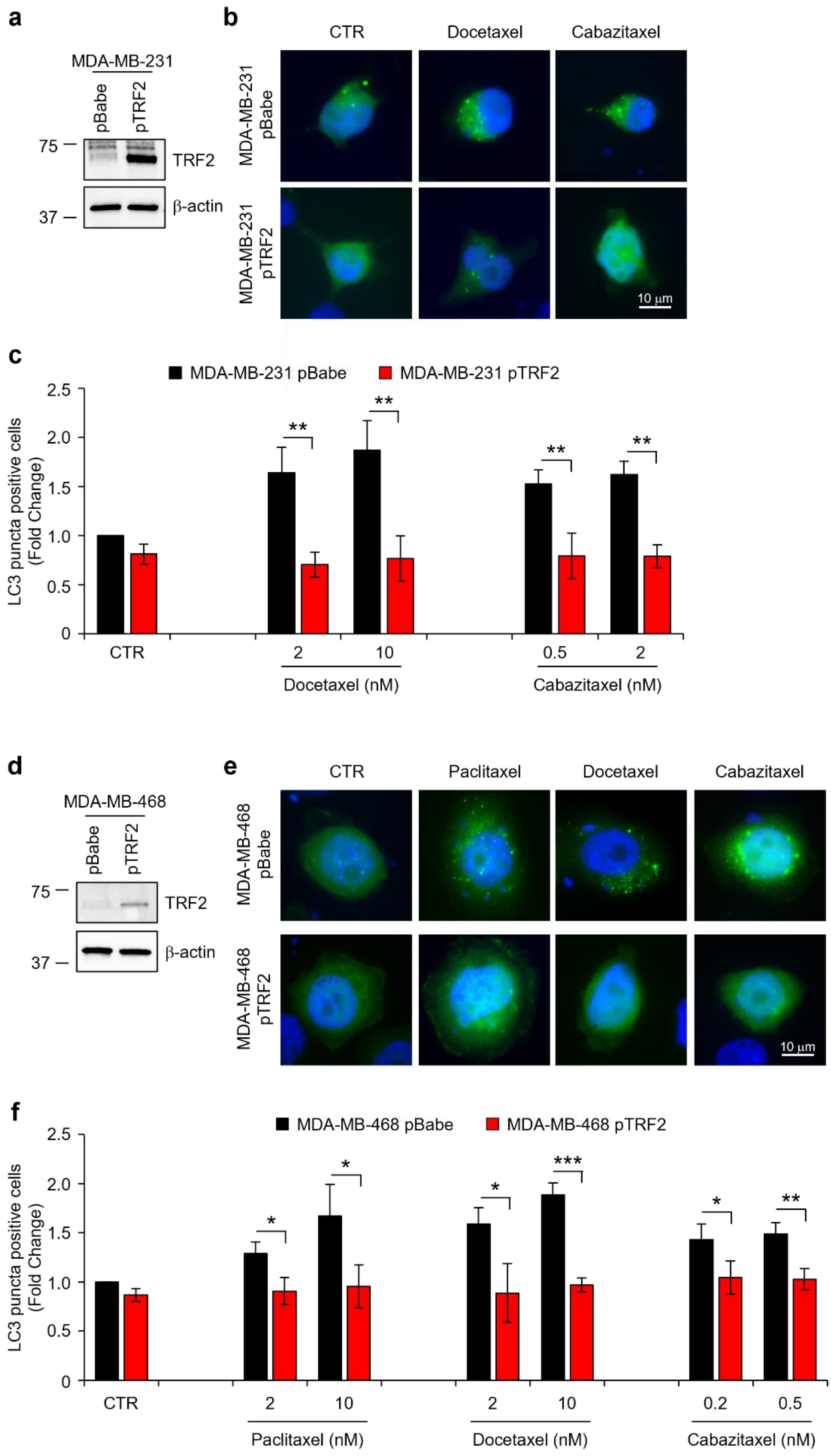

Fig. S1. TRF2 impairs the autophagic process induced by taxanes treatment in TNBC. (a) Western blot analysis of TRF2 protein levels to check cell lines. β-actin protein levels evaluation was used as

loading internal control. **(b,c)** Human triple negative breast cancer (TNBC) cell line MDA-MB-231, over-expressing (pTRF2) or not (pBabe) TRF2 were transfected with EGFP-LC3 for 24 hours and treated with Docetaxel and Cabazitaxel for 24 hours at the indicate doses. The autophagic process was evaluated by the quantitative analysis of punctate vesicular structures by fluorescence experiments. (b) Representative images of the experiment were acquired by deconvolution microscopy (63X magnification). Blue: the nuclei stained with DAPI. Green: EGFP-LC3B. (c) Histogram representing the percentage of LC3 puncta positive cells on total GFP positive cells. **(d)** Western blot analysis of TRF2 protein levels to check cell lines. β-actin protein levels evaluation was used as loading internal control. **(e,f)** Human TNBC cell line MDA-MB-468 pBabe or pTRF2 were processed for autophagic flux evaluation as in (b) e (c), respectively. (e) Representative images of the experiment described were acquired by deconvolution microscopy (63X magnification). (f) Histogram representing the percentage of LC3 puncta positive cells on total GFP positive cells.

The histograms represent the mean values ± S.D. of three independent experiments; *p<0.05, **p<0.01, ***p <0.001.

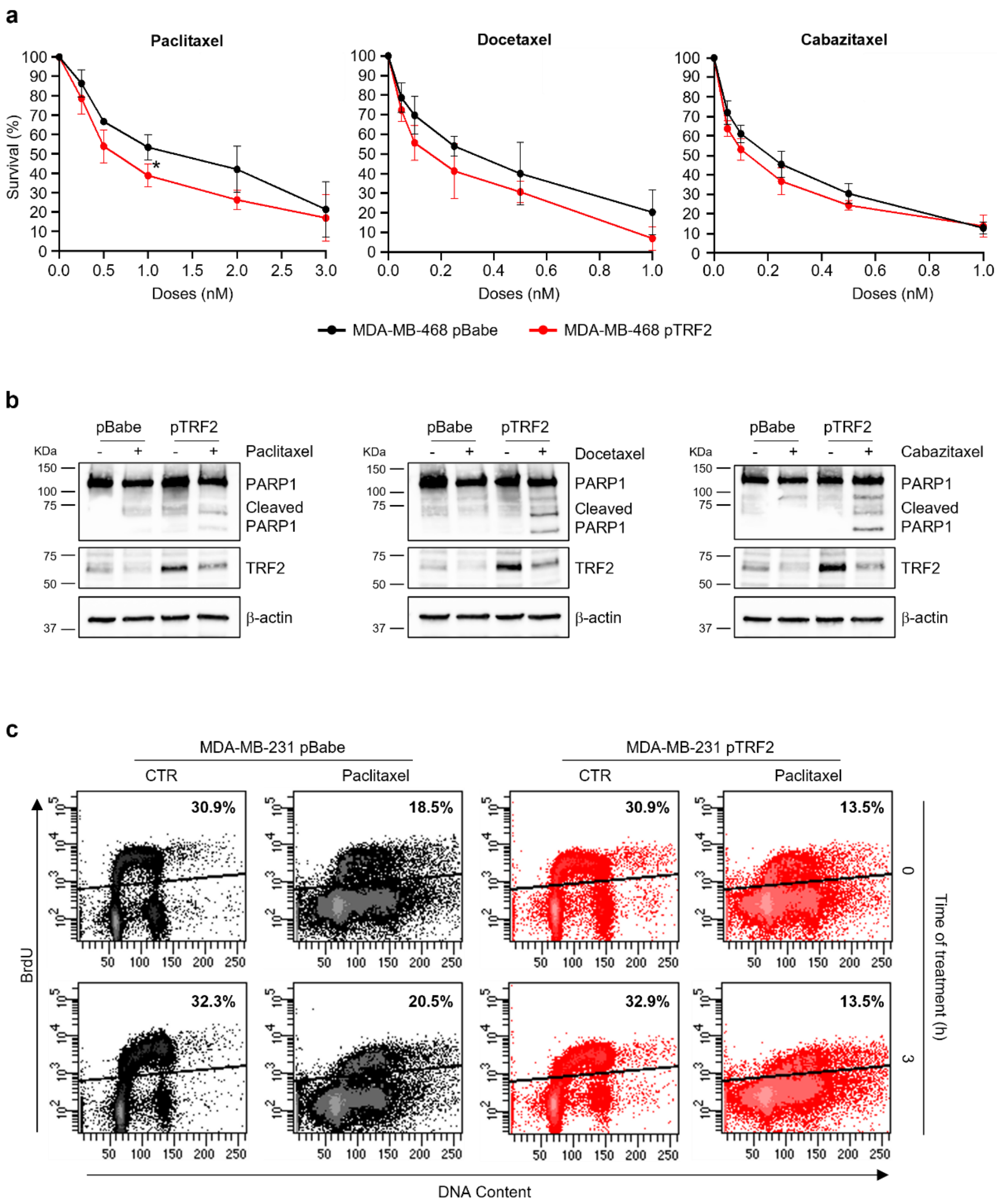

Fig. S2. TRF2 over-expression in TNBC cells confer sensitivity to taxanes. (a) Cell survival evaluation by clonogenic assay in human triple negative breast cancer (TNBC) cell lines MDA-MB-468 over-expressed (pTRF2) or not (pBabe) for TRF2 and treated with three Taxanes (Paclitaxel (a), Docetaxel (b) and Cabazitaxel (c)) at the indicated doses for 24 hours. **(b)** Western blot analysis of PARP1 and cleaved-PARP1 protein levels in human TNBC cell lines MDA-MB-231, over-expressing (pTRF2) or not (pBabe) TRF2, treated with 10 nM of Paclitaxel, 10 nM of Docetaxel and 2 nM

Cabazitaxel. **(c)** Analysis of 5-bromo-2′-deoxyuridine (BrdU) incorporation of MDA-MB-231 pBabe and pTRF2 treated with Paclitaxel 2 nM.

The graphs represent the mean values ± S.D. of at least three independent experiments. *p<0.05.

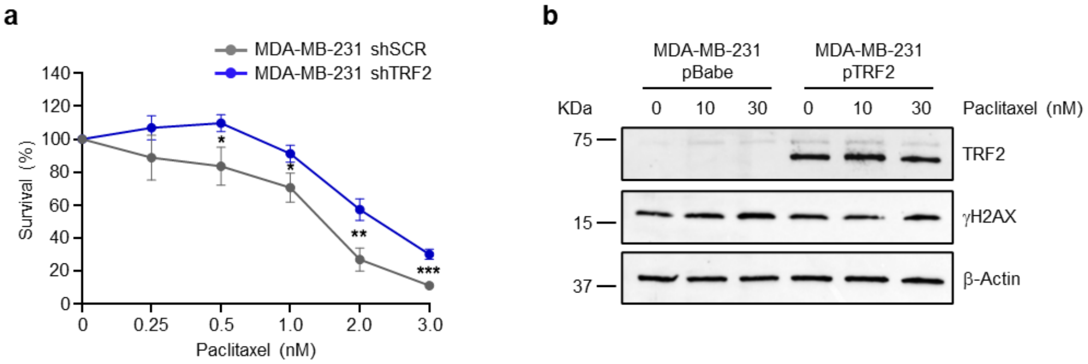

Fig. S3. Contribution of TRF2 in cancer cells sensitivity to therapy. (a) Cell survival evaluation by clonogenic assay in human triple negative breast cancer (TNBC) cell lines MDA-MB-231 silenced (shTRF2) or not (shSCR) for TRF2 expression and treated with Paclitaxel at the indicated doses for 24 hours. **(b)** Western blot analysis of protein levels of the phosphorylated form of histone H2AX, a marker of DNA damage in TNBC cells over-expressing (pTRF2) or not (pBabe) TRF2. TRF2 protein levels were used to monitor cell lines and β-actin as an internal loading control.

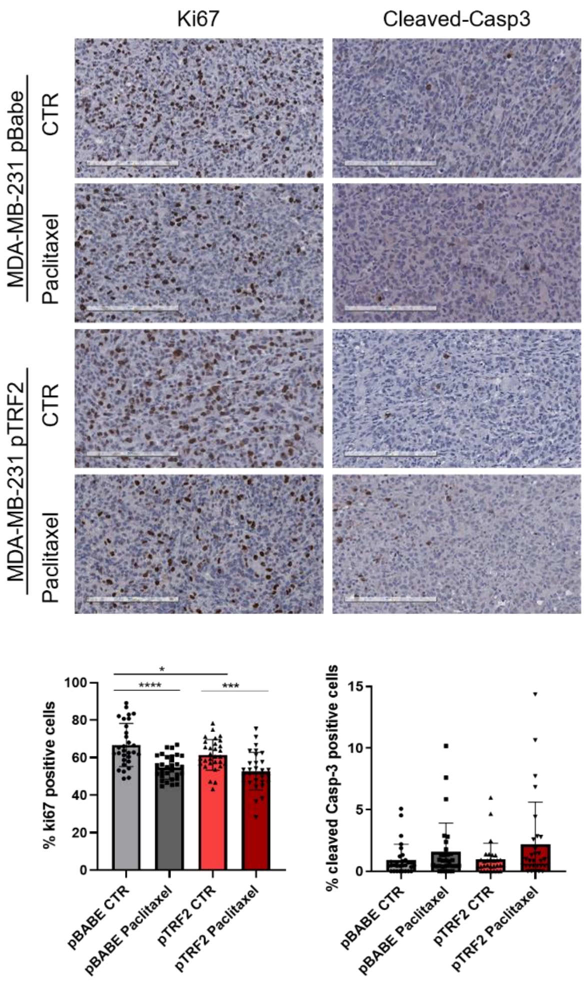

Fig. S4. Paclitaxel efficacy in xenograft TNBC tumors is enhanced by TRF2 over-expression. Histological and Immunohistochemical analysis of primary tumor established from human triple negative breast cancer cell line MDA-MB-231, over-expressing (pTRF2) or not (pBabe) TRF2, treated with Paclitaxel. *Upper panel*. Representative images of immunostained sections. *Bottom panel*. Quantification of Ki67 and cleaved-caspase 3 (expressed ad the percentage of Ki67 and cleaved-caspase 3 positive cells, respectively). Thirty fields for condition were analyzed. **p*<0.05, ****p* <0.001, *****p*<0.0001.

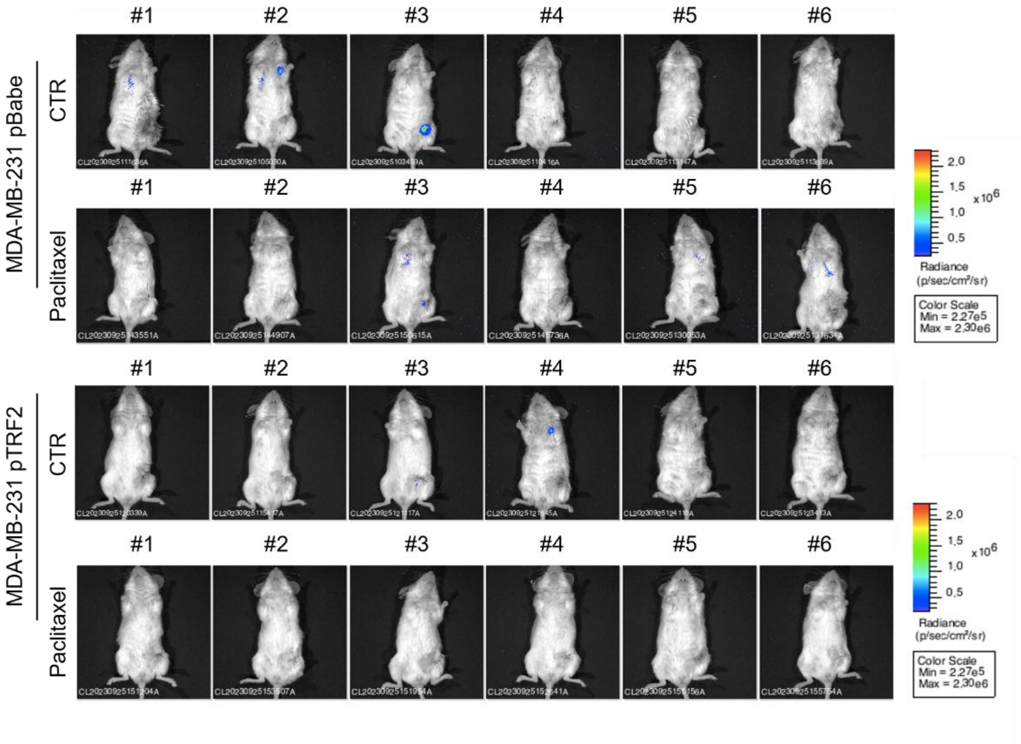

Fig. S5. Advanced orthotopic xenograft TNBC model. Fifteen days post treatment, primary tumors of mice treated as in Figure 4 A, were surgically resected and mice were analyzed by IVIS imaging to assess tumor resection. Representative pictures of six mice were shown.
